# Etymologia: *Elizabethkingia*

**DOI:** 10.3201/eid2201.ET2201

**Published:** 2016-01

**Keywords:** Elizabethkingia meningoseptica, bacteria, Elizabeth O. King

## *Elizabethkingia* [e-lizʺə-beth-kingʹe-ə]

Named for Elizabeth O. King, a bacteriologist at the US Centers for Disease Control who studied meningitis in infants, *Elizabethkingia meningoseptica* is a gram-negative, obligate aerobic bacterium in the family *Flavobacteriaceae *(Figure). King named the bacterium *Flavobacterium* (from the Latin *flavus*, “yellow”) *meningosepticum*, and in 1994 it was reclassified in the genus *Chryseobacterium* (from the Greek *chryseos*, “golden”). In 2005, it was placed in the new genus *Elizabethkingia*.

**Figure:**
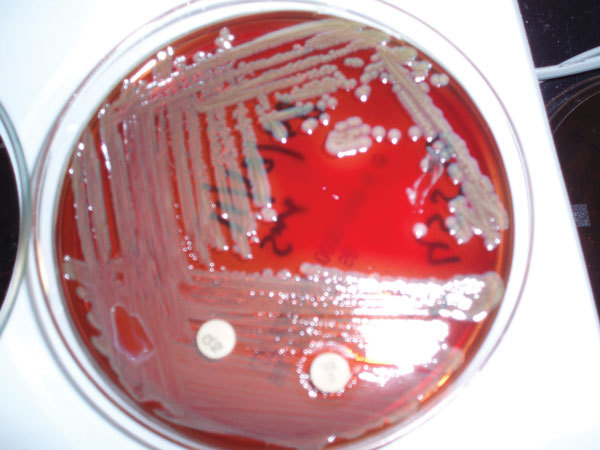
Six-day-old blood agar growth of *Elizabethkingia meningioseptica *with 5 μg vancomycin (with zone of clearing) and 10 μg colistin disks. Source: Dr. Saptarshi via Wikimedia Commons
